# Left ventricular pseudoaneurysmectomy in patient without hemodynamic instability: A case report

**DOI:** 10.1002/ccr3.6855

**Published:** 2023-01-16

**Authors:** Zviad Bakhutashvili, Lia Janelidze, Kakhaber Beria, Nana Bakashvili, Nika Kuridze

**Affiliations:** ^1^ Department of Cardiac Surgery G. Chapidze Emergency Cardiology Center Tbilisi Georgia; ^2^ Faculty of Clinical and Translational Medicine Ivane Javakhishvili Tbilisi State University Tbilisi Georgia

**Keywords:** aneurysmectomy, Dor technique, left ventricular aneurysm

## Abstract

We present a case of a 66‐year‐old male with a history of two previous diagnoses of myocardial infarction, followed by drug‐eluting stent implantation. During the check‐up, he complained of dyspnea, fatigue, and dizziness. Echocardiography revealed a massive left ventricular pseudoaneurysm (LVP). According to the patient's clinical manifestations and radiologic data, urgent surgical intervention was performed. Postoperatively, several complications appeared, which were managed successfully. The patient was discharged in stable condition. This is an interesting case of massive LVP without hemodynamic instability.

## Abstract 2

Left ventricular pseudoaneurysm can lead to serious morbidity or mortality. The reported incidence is 10%–35% and has declined, primarily after coronary angioplasty. Several treatment methods are currently available. These all need to be taken individually when surgery or medical treatment is appropriate.

## 1 INTRODUCTION

Left ventricular aneurysm (LVA) has been recognized as a serious complication of myocardial infarction (MI), which can lead to serious morbidity or death. The reported incidence of LVA after MI is 10%–35% and has declined, primarily due to treatment with coronary angioplasty performed in the acute phase of the event.^1^ A LVA is classified as true or false. A true LVA involves the entire wall thickness of the left ventricle, usually in the setting of a large transmural infarct, most commonly found in the anterior and/or apical wall. A false LVA or pseudoaneurysm occurs after myocardial rupture post‐MI, mostly 5–10 days after left circumflex artery occlusion (LCX), and is contained by adherent pericardium or fibrous scar tissue. False LVA arises commonly from the base of the inferior and/or lateral walls. The diameter of LVA varies from 1 to 8 cm. Aneurysms can be asymptomatic or cause heart failure. Several treatment methods are currently available for LVP, including drug therapy and surgical intervention. The majority of true LVA is managed conservatively, while LV pseudoaneurysms are commonly treated with urgent surgery because of the high risk of rupture.^2^ The most important surgical indication is the size of the aneurysm (>3 cm), while drug therapy can be considered in asymptomatic patients with a small one (<3 cm).^3^

## 2 CASE REPORT

**FIGURE 1:**
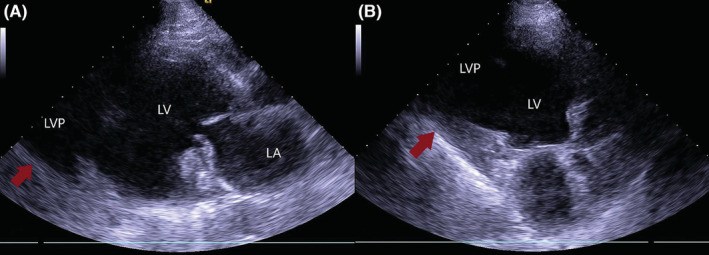
Transthoracic echocardiography showing an image of massive left ventricular pseudoaneurysm; (A) parasternal long axis view, (B) apical 4‐chamber view. LA, left atrium; LV, left ventricle; LVP, left ventricular pseudoaneurysm

**FIGURE 2:**
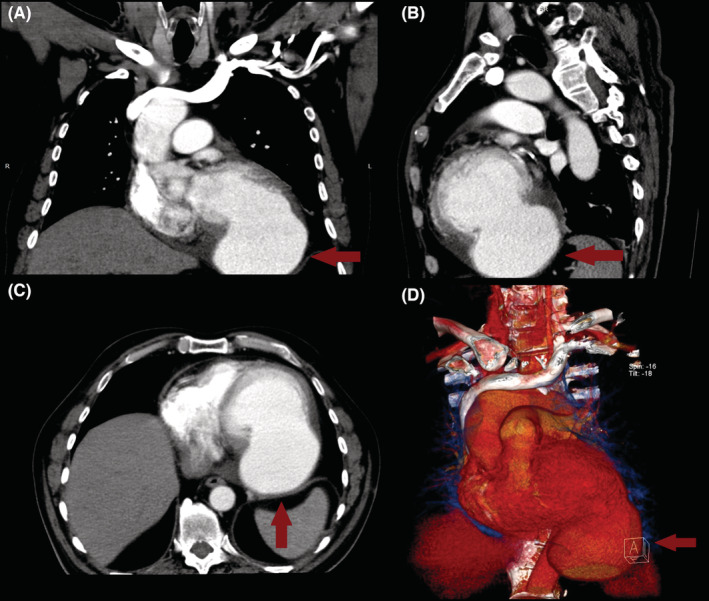
Images of chest computed tomography; (A) frontal view, (B) sagittal view, (C) axial view, (D) 3D volume rendering confirm the diagnosis of giant left ventricular pseudoaneurysm

**FIGURE 3:**
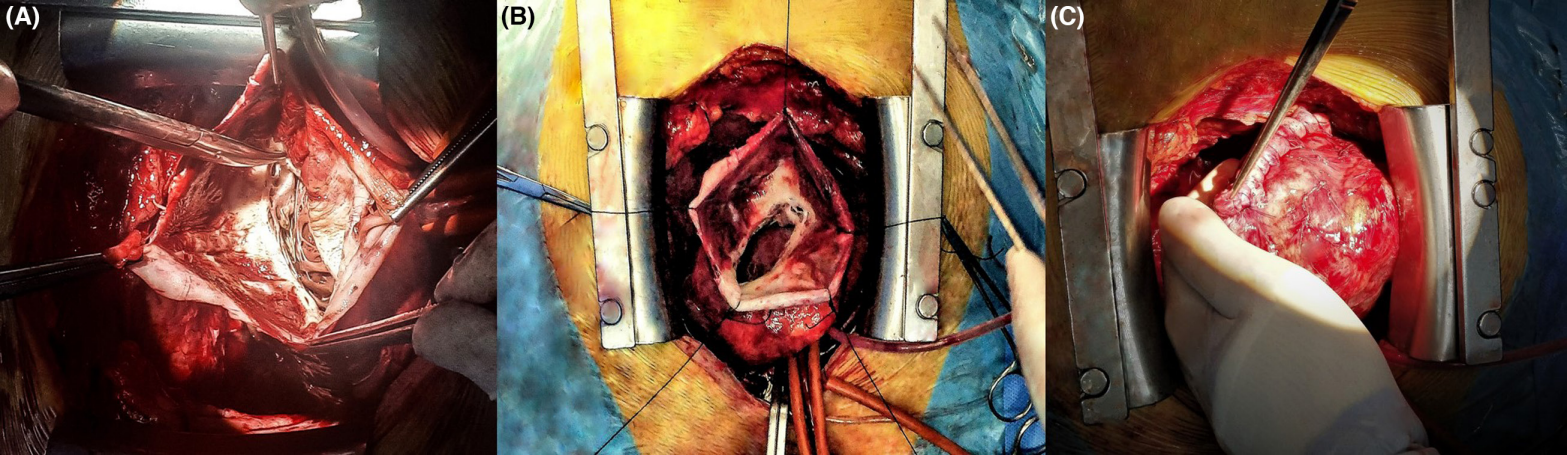
The aneurysmectomy using the Dor technique (A, B). Opened aneurysm sac with multiple scar tissue, (C) the repaired left ventricular wall

A 66‐year‐old male presented to our hospital for an outpatient visit with a history of past MI, arterial hypertension, and heart failure (NYHA III). According to the medical history, he was admitted twice to the ER for an inferior wall MI, followed by PCI (first time, two DES in the LCX, and the next time, two DES in the RCA). During his last hospitalization, he experienced atrial fibrillation. Transesophageal echocardiography (TEE) revealed reduced ventricular function (EF‐43%), left atrial dilatation of 4.2 cm without any presence of thrombus, and moderate mitral valve regurgitation. The sinus rhythm was restored after electric cardioversion. Without any significant symptoms, he was discharged from the hospital with triple anticoagulation therapy, anti‐hypertensive medications, and heart failure medications. One month after the last event, during an outpatient visit, he complained of dyspnea, fatigue, and dizziness. The ECG demonstrated sinus rhythm, and echocardiography revealed a massive left ventricular false aneurysm located in the inferolateral wall, with reduced ventricular function (EF of 32%), LVED of 175 ml, and moderate mitral regurgitation (Figure 1). A chest computer tomography (CT) with intravenous contrast demonstrated a large (8.6 × 6.8 cm) narrow‐necked aneurysm arising from the base of the inferior and lateral walls of the left ventricle (Figure 2). The patient proceeded to urgent surgery given the presence of the false aneurysm even in the absence of hemodynamic instability. The aneurysmectomy was performed using the Dor technique with endoventricular circular patchplasty, simultaneously with cardiac bypass surgery (CABG) and mitral valve annuloplasty (Figure 3). The pathological study confirmed the diagnosis (LVP). Despite the right strategy of the surgical intervention, several postoperative complications took place: cardiogenic shock, acute renal failure, bilateral pulmonary edema, bacteremia, bilateral jugular vein thrombosis, and altered neurological status due to a left‐sided subdural hematoma in the parietal lobe that did not require any surgical repair. The patient was receiving adequate medical treatment, including vasopressors, anti‐arrhythmic drugs, antibiotics, hemodialysis, and mechanical ventilation. After the successful treatment, hemodynamics, respiratory status, and laboratory tests improved. Echocardiography revealed an improved appearance of heart function. He remained upbeat and dynamic, and he was discharged home in good health.

He visits our hospital for check‐ups. Last echocardiography showed improved heart function (LVEF: 48%, LVED: 146 ml, left atrium: 3.7 cm).

## 3 DISCUSSION

A left ventricular pseudoaneurysm is a rare but very serious complication of acute MI that may lead to fatal rupture. The main pathophysiological factors are increased intracavitary pressure and structural weakness of the necrosed myocardium. The clinical presentation of LVP lacks specific characteristics. The symptoms of heart failure and associated mitral regurgitation are the most frequent presenting features.^4^ The diagnosis is based on echocardiographic data. When the diagnosis is established, most clinicians recommend urgent surgical repair. However, two critical factors must be considered when deciding between surgical intervention and conservative treatment. Pretre and colleagues suggest that asymptomatic pseudoaneurysms <3 cm in diameter might be followed conservatively,^5^ while surgical treatment is the better option for patients with symptomatic large aneurysms (>3 cm).^6^ There is, however, some evidence for medically managed hemodynamically stable LVP.^7^ It is noteworthy that a greater degree of left ventricular dysfunction, advanced age, moderate to severe mitral regurgitation, diabetes mellitus, arterial hypertension, smoking, peripheral vascular disease, and chronic renal failure have been identified as high‐risk factors for surgical mortality.^8^

In addition, a large study by Yeo and colleagues demonstrated that patients treated conservatively had higher mortality than those managed surgically.^9^ Contrary to that suggestion, Moreno and colleagues evaluated the clinical characteristics of the 10 patients with LVP (diameter ranges between 9.8 and 29.1 mm). The pseudoaneurysm was surgically repaired in only one patient presenting with hemodynamic impairment, and the remaining nine were managed conservatively because of high‐risk factors. Follow‐up showed that only two deaths occurred in medically managed patients, and others had complications such as ischemic stroke and progressive heart failure. Only conservatively managed LVP, on the other hand, can result in deterioration of left ventricular function and fatal wall rupture; however, postoperative mortality ranges from 13% to 29%.^10^ Nonfatal sequelae of conservative treatment in LVP include arrhythmias in 34%, thromboembolic and heart failure events in 29%, and recurrent MI in 22.5%.

Eventually, the LV pseudoaneurysmectomy with cardiac bypass and mitral valve annuloplasty with adequate postoperative drug treatment can effectively limit ventricular dilatation, improve left ventricular function, improve clinical symptoms, improve quality of life, and prolong survival.^11^

## 4 CONCLUSION

As noted above, the long‐term outcome of patients with post‐MI left ventricular pseudoaneurysm depends on the severity of clinical symptoms, left ventricular function, and concomitant diseases. These all need to be taken into consideration when surgery or medical treatment is appropriate.

In order to get a successful outcome in LVP management, every case should be approached individually.

## AUTHOR CONTRIBUTIONS

**Zviad Bakhutashvili:** Supervision; validation. **Lia Janelidze:** Project administration; writing – review and editing. **Kakhaber Beria:** Resources; writing – review and editing. **Nana Bakashvili:** Conceptualization; data curation; visualization; writing – original draft; writing – review and editing. **Nika Kuridze:** Conceptualization; data curation; visualization; writing – original draft; writing – review and editing.

## FUNDING INFORMATION

This work did not receive any specific grant from funding agencies in the public, commercial, or not‐for‐profit sectors.

## CONFLICT OF INTEREST

No conflict of interest.

## CONSENT

Written informed consent was obtained from the patient to publish this report in accordance with the journal's patient consent policy.

## INSTITUTIONAL REVIEW BOARD APPROVAL OR WAIVER

The manuscript has been approved by the institutional review board.

## PERMISSION TO REPRODUCE MATERIAL FROM OTHER SOURCES

No material from other sources has been reproduced.

## CLINICAL TRIAL REGISTRATION

The work is not a clinical trial.

## Data Availability

Data sharing is not applicable to this article as no datasets were generated or analyzed during the current study.
